# miRNA- and lncRNA-Based Therapeutics for Non-Hodgkin’s Lymphoma: Moving towards an RNA-Guided Precision Medicine

**DOI:** 10.3390/cancers13246324

**Published:** 2021-12-16

**Authors:** Mara Fernandes, Herlander Marques, Ana Luísa Teixeira, Rui Medeiros

**Affiliations:** 1 Molecular Oncology and Viral Pathology Group, Research Center of IPO Porto (CI-IPOP)/RISE@CI-IPOP (Health Research Network), Portuguese Oncology Institute of Porto (IPO Porto)/Porto Comprehensive Cancer Center (Porto.CCC), 4200-072 Porto, Portugal; mara.aires.fernandes@ipoporto.min-saude.pt (M.F.); ana.luisa.teixeira@ipoporto.min-saude.pt (A.L.T.); 2 Research Department of the Portuguese League against Cancer - Regional Nucleus of the North (LPCC-NRN), 4200-177 Porto, Portugal; 3 Faculty of Medicine, University of Porto (FMUP), 4200-319 Porto, Portugal; 4 Life and Health Sciences Research Institute (ICVS), School of Medicine, Campus de Gualtar, University of Minho, 4710-057 Braga, Portugal; herlandermarques@hotmail.com; 5 ICVS/3B’s-PT Government Associate Laboratory, 4805-017 Braga/Guimarães, Portugal; 6 Department of Oncology, Hospital de Braga, 4710-243 Braga, Portugal; 7 CINTESIS, Center for Health Technology and Services Research, Faculty of Medicine, University of Porto, 4200-450 Porto, Portugal; 8 ICBAS-Instituto de Ciências Biomédicas Abel Salazar, Universidade do Porto, 4050-513 Porto, Portugal; 9 Biomedical Research Center (CEBIMED), Faculty of Health Sciences of Fernando Pessoa University (UFP), 4249-004 Porto, Portugal

**Keywords:** lymphoma, Non-Hodgkin’s lymphoma, miRNAs, lncRNAs, ncRNA-based therapy

## Abstract

**Simple Summary:**

Non-Hodgkin’s lymphoma (NHL) is a very heterogenous class of hematological cancers, with variable patient outcomes. Therefore, there is an urgent need to develop new and more effective therapeutic approaches. MiRNAs and lncRNAs have emerged as the central gene expression regulators, and their deregulation has been reported to be involved in lymphomagenesis. Given their ability to simultaneously modulate multiple targets, they provide an attractive therapeutic approach to treat NHL patients. In this review, we discuss the scientific rationale behind miRNA/lncRNA-based therapies in NHL and the different targeting technologies, such as antisense oligonucleotides, CRISPR-Cas9, and nanomedicines.

**Abstract:**

Increasing evidence has demonstrated the functional roles of miRNAs and lncRNAs in lymphoma onset and progression, either by acting as tumor-promoting ncRNAs or as tumor suppressors, emphasizing their appeal as lymphoma therapeutics. In fact, their intrinsic ability to modulate multiple dysregulated genes and/or signaling pathways makes them an attractive therapeutic approach for a multifactorial pathology like lymphoma. Currently, the clinical application of miRNA- and lncRNA-based therapies still faces obstacles regarding effective delivery systems, off-target effects, and safety, which can be minimized with the appropriate chemical modifications and the development of tumor site-specific delivery approaches. Moreover, miRNA- and lncRNA-based therapeutics are being studied not only as monotherapies but also as complements of standard treatment regimens to provide a synergic effect, improving the overall treatment efficacy and reducing the therapeutic resistance. In this review, we summarize the fundamentals of miRNA- and lncRNA-based therapeutics by discussing the different types of delivery systems, with a focus on those that have been investigated in lymphoma in vitro and in vivo. Moreover, we described the ongoing clinical trials of novel miRNA- and lncRNA-based therapeutics in lymphoma.

## 1. Introduction

Non-Hodgkin’s lymphoma (NHL) is a very heterogenous group of lymphoid malignancies originating from different stages of B-cell (~90% of the cases) and T-cell or NK-cell differentiation [1]. According to the latest GLOBOCAN data, NHL represents the most common hematological malignancy worldwide, accounting for approximately 3% of cancer diagnoses and deaths [2]. The standard therapy regime for NHL remains anthracycline-containing chemotherapy (cyclophosphamide, doxorubicin, vincristine, and prednisone—CHOP), whose efficacy and patient outcome drastically improved after the introduction of the anti-CD20 agent Rituximab (R-CHOP) [3]. However, despite the improved outcomes, approximately 20–50% of patients are refractory ab initio or ultimately relapse, with only a 20–40% 2-year overall survival rate [4,5,6]. Therefore, there is an impending need for novel therapeutic approaches to replace or complement the current approaches.

For the past few years, the unveiling of the several players and molecular mechanisms involved in lymphomagenesis has permitted the development of promising new therapeutic agents that specifically target the agents and pathways involved in the malignant process [7,8]. In this instance, noncoding RNAs (ncRNAs) have emerged as important players in lymphoma pathogenesis, with several found deregulated in lymphoma, highlighting their role as potential therapeutic strategies [9,10]. Specifically, the most extensively studied ncRNA class is the microRNAs (miRNAs), characterized as small ncRNAs ~22 nucleotides in length, which act as post-transcriptional regulators of the gene expression. MiRNAs have the ability to bind to their target mRNAs, resulting in repression of the translation or target degradation [11]. Therefore, depending on their target mRNA, miRNAs can act as oncogenes (oncomiRs) or as tumor suppressor genes. Moreover, each miRNA can bind to several target mRNAs, giving them a two-faced role, underlining their potential as both direct therapeutic targets and therapeutic candidates [12]. In fact, some miRNAs have already reached clinical trials [11].

Most recently, another class of ncRNAs has come into play as an important regulator of lymphoma development, demonstrating significant clinical relevance, known as long noncoding RNAs (lncRNAs) [13]. LncRNAs represent >200-nt-long transcripts with no protein-coding capacity that can be classified as intronic, exonic, intergenic, or overlapping based on their genomic location; in sense/antisense lncRNAs based on the template strand from which they are transcribed; and in divergent/convergent lncRNAs when considering the transcription direction [14]. Contrary to the miRNAs, lncRNAs regulate gene expressions at multiple levels by interacting not only with RNA but also with DNA and proteins. LncRNAs have the capacity to modulate the chromatin structure, regulate the transcription of neighboring and distant genes, and even control RNA splicing and translation [15]. Given the broad spectrum of action, lncRNAs, with the development of nucleic acid therapeutics, open the opportunity to target and modulate a range of central pathways/processes in lymphomagenesis.

**Figure 1:**
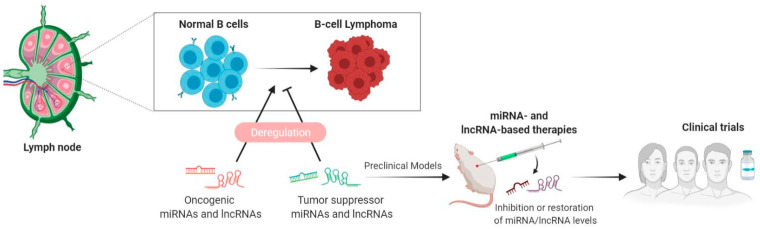
Decoding ncRNA biology from the bench to bedside in lymphoma. Deregulation of miRNAs and lncRNAs has been shown to play an important role during the process of B-cell lymphomagenesis. MiRNAs and lncRNAs can function both as oncogenes and as tumor suppressor genes. The study of altered miRNA/lncRNA expression will permit the identification of potential candidates to be use as therapeutics (to restore tumor-suppressive miRNAs/lncRNAs) or as therapeutic targets (to inhibit the levels of oncogenic miRNAs/lncRNAs) to be tested first in preclinical models and in subsequent clinical trials in lymphoma patients.

In this review, we provide a holistic overview of the current state of miRNA- and lncRNA-based therapeutics in lymphoma by addressing the different therapeutic strategies and delivery systems developed to boost their therapeutic efficacy and by reviewing the results of in vitro and in vivo studies of the therapeutic potential of miRNA and lncRNA modulation in lymphoma. Finally, we describe the current clinical trials testing the efficacy and safety of miRNA- and lncRNA-based therapies in lymphoma (Figure 1).

## 2. miRNA-Based Therapies in NHL

Acting as oncogenic or as tumor suppressors, miRNAs represent a class of master regulators of malignant transformation and progression and, thus, represent powerful candidates as therapeutics (in the role of miRNA mimics) or as therapeutic targets (in the role of antimirs). The first strategy has the rational of targeting tumor-promoting mRNAs via restoring the tumor-suppressive miRNAs in tumor cells by either using synthetic double-stranded miRNA mimics, pre-miR, or plasmid-encoded miRNA genes [11]. On the other hand, the second approach aims to inhibit the levels of oncomiRs, which are frequently overexpressed in cancer, allowing the restoration of the expression of tumor-suppressor targets. Different methodologies of oncomiR inhibition are being developed by using single-stranded antisense, anti-miR oligonucleotides (AMOs), locked nucleic acid (LNA) anti-miRs, antagomiRs, miRNA sponges, and small molecule inhibitors of miRNAs (SMIRs) [11,16,17,18,19,20].

While the theory behind miRNA-based therapy is somehow straightforward, the challenges of this approach reside in its delivery. The presence of multiple ribonucleases and reticuloendothelial system clearance in the blood make miRNAs unstable in the circulation. Moreover, unmodified miRNA antagonists and miRNA mimics are unable to cross the cell membrane or the vascular endothelium due to their negative charges [21]. The efficacy of miRNA delivery depends also on blood perfusion in tumors and cell-specific delivery. Not only the tumor microenvironment, tumor-associated immune cells especially can nonspecifically uptake and capture miRNAs, but also, it is essential to prevent the disruption of the healthy tissue [21,22,23]. Therefore, to overcome these obstacles, numerous miRNA delivery methodologies are being developed, both local and systemic delivery strategies (Table 1).

Despite the difficulty of getting bench-based microRNAs to the bedside, several companies are developing miRNA-based drugs, some of which are already being tested in phase I and phase II clinical trials. In fact, in 2018, the FDA approved the first siRNA drug, Patisiran, to be employed in the treatment of a rare polyneuropathy caused by transthyretin-mediated amyloidosis [24,25,26].

**Table 1:** Summary of the miRNA mimics or inhibitors tested in vivo.

Chemical Modifications	Delivery Systems	miRNAs	Target Strategy	Delivery Route	Target Disease	Ref.
	Lipid-based	miR-34a	Restoration	Intratumoral Tail vein	DLBCL	[27]
	Lipid-based			Subcutaneous	MM	[28,29]
	Viral-based					
LNA		miR-155	Inhibition	Tail vein	Waldenstrom macroglobulinemia	[30]
PNA	Peptide-based			Intravenous	B-cell lymphoma	[31]
PNA	Polymer-based			Intravenous Intratumoral		[32]
	Viral-based	miR-15a/16	Restoration	Intravenous Intraperitoneal	CLL	[33]
	Viral-based	miR-144/451	Restoration	Subcutaneous	B-cell lymphomas	[34,35]
	Viral-based	miR-181a	Restoration	Subcutaneous	DLBCL	[36,37]
	Viral-based	miR-27b	Restoration	Subcutaneous	DLBCL	[38]
	Lipid-based	miR-28	Restoration	Intratumoral	BLDLBCL	[39]
	Viral-based			Intravenous		
	Lipid-based	miR-21	Inhibition	Subcutaneous	MM	[40]
	Viral-based	miR-17∼92 cluster	Inhibition	Intratumoral	DLBCL	[41]
2′ O-methyl-group	EV-based	miR-125	Inhibition	Intraperitoneal	AML	[42]

Abbreviations: LNA: locked nucleic acid, PNA: peptide nucleic acids, DLBCL: diffuse large B-cell lymphoma, MM: multiple myeloma, CLL: chronic lymphocytic leukemia, BL: Burkitt lymphoma, and AML: acute myeloid leukemia.

### 2.1. Local Delivery

Intratumoral injection or local administration of miRNA mimics or inhibitors has shown effective gene silencing and antitumoral effects, with reduced nonspecific uptake by normal healthy tissue and reduced toxicity and immunogenicity compared with systemic delivery. However, the local delivery of miRNAs is limited to localized and readily accessible primary tumors such as melanoma, breast cancer, or cervical cancer. The therapeutic potential of miR-34a replacement therapy was shown in a xenograft model of DLBCL, where the intratumoral administration of synthetic miR-34a mimics resulted in tumor growth inhibition [27]. Trang et al., using an aggressive human non-small cell lung cancer (NSCLC) xenograft model, showed that the intranasal delivery of a lentiviral vector expressing let-7a resulted in an increased expression of let-7 in the lungs and the subsequent growth inhibition of KRAS-dependent lung tumors. Moreover, locally polymer-based delivered let-7b led to a 60–70% reduction of the tumor burden [43]. Sureban et al. developed a nanoparticle-mediated intratumoral delivery of DCAMKL-1-specific siRNA, capable of inducing let-7a and miR-144 expression, which, in turn, repressed proto-oncogene c-Myc and Notch-1 in colorectal cancer xenografts, resulting in tumor growth inhibition [44].

Despite the advantages of the local delivery of miRNAs, this type of approach is primarily limited by the tumors’ location and stage. Therefore, the development of systemic delivery systems is essential to broaden the spectrum to other types of cancers and metastatic cancers. To improve the binding affinity, stability, and target modulation, two converging strategies are applied: chemical modifications of miRNAs and the development of delivery vehicles to encapsulate miRNAs.

### 2.2. Systemic Delivery

**Figure 2:**
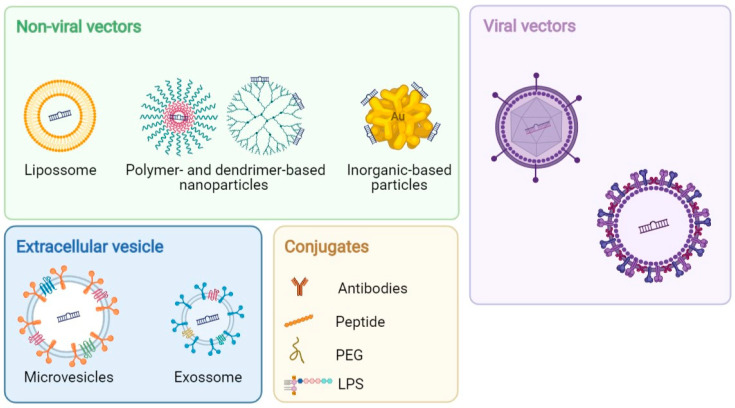
Schematic representation of the commonly used and emerging nanoplatforms for ncRNA delivery. (Abbreviations: PEG: polyethylene glycol and LPS: lipopolysaccharide).

Advances in the chemical modifications of miRNAs, such as the addition of a 2′-O-methyl group; locked nucleic acid (LNA) oligonucleotides; peptide nucleic acids (PNAs); phosphorothioate-like groups; and cholesterol-, biotin-, and amino-modified oligonucleotides, are being investigated. The addition of a 2′-O-methyl or 2′-O-methoxyethyl group to ribose was shown to enhance the binding affinity and stability of anti-miRNA while efficiently and specifically silence the targeted endogenous miRNAs in numerous tissues, such as the bone marrow [19,45]. LNA-mediated anti-miR-155, targeting the seed region of miR-155, showed a significant inhibition of cell proliferation of low-grade B-cell lymphomas in vitro and decreased the tumor burden in a xenograft mouse model of Waldenstrom macroglobulinemia (WM) [30]. Cheng et al. showed that miR-155 oncomiR, one of the most extensively studied miRNAs for its therapeutic potential, can be silenced by linking PNA anti-miR-155 to a peptide with a low pH-induced transmembrane structure (pHLIP). This conjugation efficiently inhibited the tumor growth and increased mouse survival in a mouse model of lymphoma [31]. LNA anti-mir-122 systemic administration was shown to downregulate, in a dose-dependent manner, liver-specific miR-122, which prevents hepatitis C virus (HCV) replication. The promising results of the LNA anti-mir-122 drug as a preventive therapy for HCV-induced hepatocellular carcinoma (HCC) led to phase II trials for the treatment of HCV infection [46]. Despite the chemical modifications, modified miRNAs have reduced tumor uptake and biodistribution due to rapid renal and hepatic clearances, which results in short half-lives [21]. Therefore, several viral and nonviral vectors are being developed as miRNA delivery systems (Figure 2).

### 2.3. Vectors-Based Delivery Systems

#### 2.3.1. Viral Vectors

Viral vectors, such as lentivirus, adenovirus, and adeno-associated virus (AAV), can carry and deliver miRNA mimics or antagonists to the nuclei of tumor cells. Moreover, the conjugation of targeting moieties to viral capsid proteins by genetic manipulation allows specific delivery into the tumors by enhancing the affinity between viral vectors and cancer-specific receptors [21]. In a study by Kasar et al., the systemic lentiviral delivery of miR-15a/16 restored the expression of these miRNAs in a New Zealand Black (NZB) mouse model of CLL [33]. The viral-mediated delivery of miR-144/451 restored their expression, resulting in the growth inhibition of a B-cell line xenograft in vivo [34,35]. Similarly, the viral-mediated restoration of miR-181a (downregulated in human DLBCL) and miR-27b (downregulated in human DLBCL and splenic marginal zone lymphoma (SMZL)) resulted in the growth inhibition of a human DLBCL-cell line xenograft [36,37,38]. The restoration of miR-28 by both viral vectors or as synthetic, clinically amenable molecules was shown to inhibit tumor growth in human Burkitt (BL) and DLBCL xenografts and in a primary BL murine model after intratumor or systemic administration [39]. Lentivirus-based miR-34a replacement or miR-34a synthetic mimics induced growth inhibition and apoptosis in multiple myeloma (MM) cells in vitro and exerted a powerful antitumor activity in MM xenografts in SCID mice and in a SCID-synth-hu model [29]. Su et al. demonstrated the potential of simultaneous targeting multiple oncomiRs, which are usually upregulated in DLBCL but not in normal cells, as a therapeutic strategy for B-NHL. In this study, they used a synthesized interfering long noncoding RNA (i-lncRNA) that simultaneously inhibited 13 oncomiRs, including five miR-17~92 cluster miRNAs, by competing with the corresponding target mRNAs for binding oncomiRs. Moreover, the treatment approach involving adenovirus-mediated i-lncRNA expression was shown to significantly inhibit human DLBCL xenograft growth [41]. Despite their common use due to their high efficiency, viral-based miRNA delivery systems are still associated with high immunogenicity, toxicity, and size limitations, which impose a serious obstacle to clinical applications. Therefore, nonviral vectors, such as lipid, polymer, inorganic, and extra-cellular vesicle carrier-based approaches, are rising as preferred alternatives for research and clinical studies.

#### 2.3.2. Nonviral Vectors

Lipid-based nanoparticles are the most frequently used nanodelivery systems because of their easy synthesis, high stability, loading efficiency, low immunogenicity, and versatility of administration routes [47]. To date, there have been several commercially available cationic liposomes that have been routinely used for miRNA delivery, such as Lipofectamine^®^ (Invitrogen, Carlsbad, CA, USA), TransIT^®^ 2020 (Mirus Bio LLC, Madison, WI, USA), SiPORT™ (Invitrogen, Carlsbad, CA, USA), SilentFect™ (Bio-Rad Laboratories, Inc. Hercules, CA, USA), and Oligofectamine™ (Invitrogen, Carlsbad, CA, USA) [48]. The main obstacle that limits the clinical application of cationic lipids is their low delivery efficiency in vivo. To overcome this problem, several new methods for synthesizing lipid nanocomplexes have been developed. For example, the development of neutral liposomes and the conjugation of a polyethylene glycol (PEG) functional group to cationic lipids prevents phagocytosis and prolonged circulation, thus enhancing the overall efficacy [20]. The administration of a lipid-based miR-34a mimic, either intratumorally or systemically (using a neutral lipid emulsion (NLE)), resulted in a 95% and 76% reduction in tumor growth, respectively, in a DLBCL mouse model [27]. MiR-28a-5p, whose expression is frequently reduced in human B-cell neoplasia and associated with the downregulation of downstream BCR-signaling effectors, such as PI3K and AKT, has shown therapeutic potential as a replacement therapy for B-NHL. The administration of synthetic miR-28a-5p mimicked using a liposome delivery approach, impaired proliferation and survival of lymphoma cells, and abrogated tumor growth in MD901 DLBCL and Ramos human BL xenograft mouse models and in a λ-MYC transgenic mouse BL model [39]. Conversely, the lipid-based delivery of miR-21 inhibitors significantly inhibited tumor growth in a human MM xenograft model [40]. More recently, several approaches have emerged to enhance the targeted liposome-based miRNA delivery to specific cells. For example, Di Martino et al. synthesized a stable nucleic acid lipid particle (SNALPS) carrying miR-34a, which showed high-vesicle loading, good transfection efficiency, and stability in the serum. Moreover, SNALPS-mediated miR-34a delivery efficiently inhibited MM cell growth in vitro and in vivo, confirming the high potential of this carrier in miRNA-based therapy [28].

Polymer-based delivery methods primarily use polyethylenimine (PEI), which results from the conjugation of positively charged amine groups with an anionic RNA, preventing RNA degradation and promoting cellular uptake and intracellular release [49]. However, the use of PEIs has been limited in the current clinical research due to their low transfection efficiency and cytotoxicity. The use of other polymers, such as PEG, a nonionic and hydrophilic polymer covalently fused to PEI, can reduce toxicity by improving its biocompatibility. Avci et al. proved that PEG/PEI nanocomplex polymeric vectors improved the stability and transfection efficiency of miR-150 in human leukemia cells [50]. Another approach that has been employed is the FDA-approved biomaterial poly(lactide-co-glycolide) (PLGA), a copolymer of poly lactic acid (PLA) and poly glycolic acid with a well-documented utility for sustained drug release and clinical use. The systemic delivery of PNA anti-miR-155 conjugates encapsulated in PLGA polymer nanoparticles efficiently inhibited miR-155, which, in turn, resulted in reduced tumor growth in vivo, suggesting a therapeutic potential in B-cell tumor models [32].

The advancements in nanotechnology have led to the development of various inorganic compound-based nanoparticles as excellent nanocarriers for miRNA delivery both in vitro and in vivo. Although there is a lack of studies when compared to other types of vectors previously discussed, the majority of studies have focused mainly on gold, Fe3O4-based, and silica-based nanoparticles [51,52,53]. Inorganic compound-based delivery systems have received attention specially due to their high bioactivity, biocompatibility, and chemical stability in vivo [54]. Gold nanoparticles (AuNPs) have shown low cytotoxicity and immunogenicity, and given their physicochemical, optical, and electronic properties, they have been considered as an excellent nonviral miRNA delivery system. A study by Ghosh et al. demonstrated that PEG-conjugated AuNPs are able to successfully deliver miR-1 to cancer cells, showing a high transfection efficiency and low cytotoxicity [55]. Moreover, a multifunctional AuNP was synthesized to simultaneously deliver three anticancer agents—AS1411, doxorubicin, and anti-miR221—to drug-resistant leukemia cells. These nanoparticles were able to induce the miR-221-mediated reduction of cell proliferation and clonogenic potential, induce apoptosis, and sensitize drug-resistant cells, enhancing the chemotherapy efficacy [56].

In recent years, since the discovery of extracellular vesicles (EVs) as natural carriers of biomolecules like miRNAs involved in cell-to-cell communication, the exploitation of EVs for therapeutic applications has been under study. The intrinsic characteristics of EVs, including stability in circulation and biocompatibility, as well as low immunogenicity and toxicity, render them attractive miRNA delivery vehicles. Moreover, the manipulation of their various membrane ligands allows targeted cargo delivery to specific cells and tissues [57]. Usman et al. showed that blood cell-derived EVs carrying anti-miR-125b AMOs efficiently downregulated miR-125 in acute myeloid leukemia (AML) cells in vitro and effectively suppressed leukemia progression in a mouse model [42].

Despite their efficacy and promising potential as a delivery vehicle in conjunction with the targeting ligand on their surfaces, the mass production of EVs and effective packaging methods remain a challenge.

### 2.4. Targeting of miRNAs via CRISPR/Cas9

In the past few years, the development of a CRISPR–Cas9 system unveiled a world of new opportunities for the therapeutic targeting of not only coding but also noncoding genes [58]. The CRISPR/Cas9 system has emerged as an excellent option for miRNA therapeutic inhibition. This state-of-the-art genome editing tool permits the inhibition of miRNA expression by targeting their biogenesis sites, resulting in a functional knockout [59]. Specifically, the CRISPR-Cas9- mediated inhibition of miRNA can be achieved by introducing indels (insertions and deletions) in the terminal loop or 5′ region of pre-miRNA, which disrupts Drosha processing [60,61]. Moreover, Chang et al. also reported miRNA knockouts by targeting sequences within/adjacent to Drosha and Dicer processing sites in the secondary stem–loop structures of primary miRNA, which are crucial for processing miRNA biogenesis [62]. In this study, Chang et al. not only demonstrated efficient and specific decreases in mature miRNA levels in vitro with the minimized crossing off-target effects among miRNA members of the same family or those with highly conserved sequences but, also, the in vivo long-term stability of CRISPR/Cas9 miRNA knockdown for up to 30 days [62].

Interestingly, CRISPR/Cas9 technology permits not only the inhibition of a single miRNA but also the simultaneous inhibition of multiple miRNAs. Narayanan et al. performed a CRISPR/Cas9 mutagenesis strategy to abrogate the activity of an entire miRNA family. They screened 45 mutations in 10 miRNA genes both in silico and in vivo and demonstrated that 99% of CRISPR/Cas9 mutations altered the critical sequences within each hairpin primary miRNA structure, blocking the recognition by miRNA biogenesis machinery and thus inhibiting the miRNA family expression in vivo [63].

Despite the recent research regarding the promising utility of CRISPR/Cas gene editing technology as a cancer therapeutic approach, there is still a lack of information when it comes to targeting miRNAs compared to targeting coding genes.

## 3. MicroRNA-Based Combinatorial Cancer Therapy

Although NHL patients may initially respond to treatment, most patients relapse within the first 2 years after the initial treatment and often develop chemotherapy resistance [64,65]. The acquisition of drug-resistant mutations in tumor cells due to selective pressure ultimately results in the limited efficacy of cancer therapies [66].

The combination of different therapies, targeting different mechanisms, has been proposed as an option to overcome therapeutic resistance. Given the ability of miRNAs to target multiple genes of different resistance-mediating pathways, the modulation of their levels is attractive as miRNA-based combinatorial cancer therapy. The rationale of this approach is to use miRNA-based therapy to sensitize cancer cells to other anticancer therapies. To date, studies have identified in vitro various miRNAs involved in the modulation of human lymphomas cell sensitivity to chemotherapy [67,68,69,70,71]. Leivonen et al. demonstrated that the human shMIMIC lentiviral-mediated transduction of miR-370-3p, miR-381-3p, and miR-409-3p in SU-DHL-4 cells resulted in the downregulation of the phosphatidylinositol (PI), MAPK, and BCR-signaling pathways, which, in turn, enhanced the chemosensitivity of DLBCL cells to rituximab or doxorubicin in vitro [71]. Another study showed that miR-34a-5p is also associated with the chemosensitivity of lymphoma cells. The lentivirus transduction of miR-34a-5p resulted in the decreased cell viability of human DLBCL cell lines treated with doxorubicin, which seems to be associated with the downregulation of FOXP1 [69]. Enhanced drug sensitivity was also observed in human DLBCL cell lines treated with miRNA mimics of miR-197, miR-199a-3p, miR-497-5p, and miR-187, exhibiting increased apoptosis and decreased cell viability when combined with doxorubicin or vincristine treatment [68,70,72]. In the study of Tian et al., the transfection of a miR-497 mimic into human MM cell lines enhanced the bortezomib treatment efficacy, shown by the reduction of cell viability in addition to the cell cycle arrest and reduced colony formation [73]. 2′-O-Methylation-modified hsa-miR-324-5p potentiates the anti-MM activity of bortezomib in vitro and in vivo by inhibiting MDR proteins and regulating BCL2 family gene expressions [74]. Conversely, the inhibition of miR-21 and miR-155 by miRNA inhibitors significantly increased the cytotoxic effect of the CHOP regime or doxorubicin and rituximab, respectively, in human DLBCL or BL cell lines. Moreover, the modulation of drug resistance by these miRNAs seems to be associated with the downregulation of MDR1 in the case of miR-21 and LMP1 for miR-155, both involving the activation of the PI3K/AKT/mTOR pathway [67,75,76]. A study performed by Sun et al. demonstrated a miR-148b mimic-induced sensitivity to CHOP using human DLBCL xenografts in mouse models, and this effect seems to be associated with Ezrin downregulation [77]. Moreover, miR-148b has been also associated with modulation of the radiation response by NHL cells. Upregulated miR-148b by miR-148b mimic transfection resulted in significantly enhanced cell death compared to the controls, which the authors suggested could be due to the promotion of radiation-induced apoptosis by miR-148b [78]. Following the same line of thought, both in vitro and in vivo studies by Wu et al. demonstrated that an induced miR-150 expression by lentiviral-mediated transfection in NK-/T-cell lymphoma cells enhances the radiosensitivity by inhibiting the AKT pathway [79].

Even though several studies have proven the potential of miRNA-based combinations as a way to enhance the current cancer therapy efficacy, further investigations are needed, especially concerning the selection of miRNAs that are particularly relevant to the specific cancer type and associated with the therapy-resistant pathways.

## 4. LncRNA-Targeted Therapeutics

Despite the tissue- and cell-specific expression characteristics of lncRNAs, making them attractive therapeutic targets, only recently have they become the focus of investigations. Similar to miRNA-targeting approaches, lncRNAs can be targeted by different methods based on their potential function. The knockdown of oncogenic lncRNAs can be achieved by using a siRNA strategy that induces a dicer- and argonaute-dependent cleavage or by using chemically modified ASOs that target the lncRNAs for RNase H-dependent degradation. Alternatively, lncRNA transcription can be modulated by steric blocking the gene promoter or by performing genome-editing techniques, such as CRISPR/Cas9. The last approach to target lncRNA functions is by steric block lncRNA–protein interactions or by inhibiting the formation of a secondary structure via ASOs.

The previously mentioned chemical modifications and delivery strategies of miRNA-based therapeutics are also applied in lncRNA-based therapy in order to increase the stability and specificity and to increase the intracellular uptake in vivo [80].

### 4.1. LncRNA-Targeting by Nucleic Acid Therapeutics

Presently, there are two major strategies of nucleic acid therapeutics to target lncRNAs based on double-stranded RNA-mediated interference (RNAi) and single-stranded ASOs.

An RNAi-targeting strategy is essentially based on the use of small interfering RNAs (siRNAs), which transiently target the lncRNA of interest, and short hairpin RNAs (shRNAs), which are stably expressed. The in vitro knockdown of lncRNAs by RNAi has been performed successfully in numerous cell lines, including lymphoma cell lines. For example, the siRNA-induced knockdown of MALAT1 in mantle cell lymphoma (MCL) cell lines has resulted in decreased cell viability and colony formation, increased cell apoptosis, and cell cycle arrest at the S/G1 transition [81]. However, the in vivo inhibition of lncRNA using siRNA is more challenging, especially due to inefficient delivery systems and a lack of bioavailability of siRNAs in animals [82]. Guo et al. used BALB/c nude mice to determine the effect of lncRNA MCM3AP-AS1 on cell proliferation and tumor growth. The group observed that MCM3AP-AS1 knockdown improved lymphoma sensitivity to doxorubicin by inhibiting cell proliferation and enhancing apoptosis in vitro and resulted in reduced tumor growth in vivo [83]. Cheng et al. achieved TUG1 knockdown by transfecting lymphoma cells with siRNAs using lipid-based delivery, which resulted in decreased DLBCL cell growth and promoted apoptosis in vitro. Moreover, the subcutaneous injection of si-TUG1 cells in nude mice resulted in a dramatic decrease of tumor growth and lung metastatic nodules [84].

However, the efficiency of the RNAi approach has been questioned, specifically regarding the susceptibility of nuclear- and enhancer-associated lncRNAs to RNAi machinery, predominantly located in the cytoplasm. To overcome this obstacle, usually, several siRNA sequences are screened to determine the more effective path to knock down a specific lncRNA [85].

Alternatively, ASOs or gapmers, which are synthetic chimeric ASO-containing LNA/DNA mixmers, use a DNA/RNA hybrid strategy to target RNA by using the base pairing rules. Compared to RNAi, ASOs were shown to be more efficient in knocking down nuclear lncRNAs while still efficient for cytoplasmatic lncRNAs, whereas cytoplasmatic lncRNAs are more efficiently silenced by RNAi [85]. Additionally, the ASO-based approach has been proven successful in both preclinical studies (animal models) and human clinical trials, with ASOs being recently approved by the FDA for clinical use in neurodegenerative diseases [86].

The LNA-gapmeR-mediated knockdown of NEAT1 in MM cells resulted in the inhibition of cell proliferation while triggering apoptosis in vitro and antitumor activity in in vivo in a murine MM model with optimal tumor uptake and without systemic toxicity. Moreover, LNA-gapmeR treatment enhanced the drug sensitivity of MM cells to anti-MM drugs, revealing a synergistic effect [87].

The major drawbacks of both RNAi and ASO strategies remain the incomplete and transient knockdown of lncRNAs and unpredictable off-target effects, which represent critical obstacles for clinical application [80].

### 4.2. Targeting of lncRNA-Expressing Loci via CRISPR/Cas9

The recent advances in genome editing technology such as CRISPR/Cas9 provide a powerful alternative method for transcriptional silencing lncRNAs both in vitro and in vivo.

Loss-of-function of a specific lncRNA can be achieved, for example, by a CRISPR deletion (CRISPR-del) approach or CRISPR inhibition (CRISPRi). CRISPR-del provides perhaps the most straightforward approach, in which CRISPR–Cas9 complexes induce double-stranded breaks at sites lacking lncRNA loci, and by the nonhomologous end-joining process, the target fragment is removed [88]. The most efficient manner to silence lncRNAs is by deleting small regions containing the promoter and transcriptional start site (TSS) [89]. For example, David et al. used CRISPR-mediated deletion of the CRNDE locus in MM cells and observed a decrease in cell proliferation and adhesion, increased dexamethasone sensitivity, and reduced tumor growth in vivo [90].

Alternatively, the most preferable method is the transcriptional repression of lncRNA by CRISPRi. This methodology uses a catalytically inactive Cas9 fused to transcriptional repressors in which the resulted fusion protein is the target of a specific gene promoter by an RNA-guided targeting platform [91]. In this instance, Raffeiner et al. used CRISPRi to target MYC-regulated noncoding genes in human B cells and successfully identified 320 noncoding loci that are involved in cell growth in human lymphoid cell lines. Moreover, the transcriptional repression of any of the selected lncRNAs diminished cell proliferation [92].

The major disadvantages of CRISPR-Cas9 targeting lncRNAs are the complex architecture of the genomic loci bordering different lncRNA genes, which may lead to the disturbance of neighboring or overlapping genes, leading to false-positive phenotypic changes. In fact, a study by Goyal et al. showed that only 38% of 15,929 identified lncRNA loci could be safely manipulated by CRISPR applications [93].

### 4.3. Small Compounds Targeting lncRNAs

Lastly, lncRNA silencing can be achieved by interfering with secondary and tertiary structures of specific lncRNAs using small molecules. The premise of this therapeutic approach is to disrupt the lncRNA spatial structure or block lncRNA–protein interactions. With this in mind, Pedram Fatemi et al. developed a tool to quantify lncRNA–protein interactions and identified small molecules with the ability to modulate their interactions. Based on their analysis, they reported that a small compound known as ellipticine could inhibit the interaction between BDNF-AS–EZH2 and HOTAIR–EZH2 [94]. Another identified druggable lncRNA was MALAT1, which presented a triple-helix structure at the 3ʹ end. Abulwerdi et al. used a small molecule microarray strategy and identified multiple MALAT1 ENE triplex-binding compounds capable of modulating MALAT1 downstream genes [95]. Another interesting example was demonstrated by Mercatelli et al. in their study in which HULC downregulation, using small-molecule YK-4-279, resulted in a reduction of TWIST1 expression by unleashing miR-186 and permitting its binding to TWIST1 [96].

## 5. Clinical Trials for miRNA and lncRNA-Based Drug Candidates

Given the promising preclinical results, some miRNA-based drug candidates are currently in phase I and phase II clinical trials for the treatment of diverse pathologies, including cancer (Table 2). Mirna Therapeutics, Inc. (Carlsbad, CA, USA) developed the first miRNA-based cancer therapy to enter clinical trials, MRX34, a liposome-encapsulated miR-34 mimic. The clinical trial (NCT01829971 [97]) included patients diagnosed with primary liver cancer, NSCLC, lymphoma, melanoma, multiple myeloma, or renal cell carcinoma to evaluate the safety of MRX34. However, due to severe immune-related adverse responses in five patients, the FDA terminated the phase Ib study. The cause of the severe adverse effects remains unclear but is now under investigation in preclinical trials [98]. A phase I clinical trial (NCT02369198 [99]) using an epidermal growth factor receptor (EGFR) antibody-coated bacterial-derived minicell system loaded with miR-16 mimic (MesomiR-1) showed an effective inhibition of tumor growth. During this trial, MesomiR-1 was intravenously administered to 26 patients with malignant pleural mesothelioma or non-small cell lung cancer (NSCLC) refractory to the standard therapy to determine the maximum tolerated dose, allowing the initiation of a phase II clinical trial [100]. Based on the results of this trial, additional studies are needed to investigate the antitumor activity of TargomiRs in combination with chemotherapy or immune checkpoint inhibitors [101]. MiRagen Therapeutics, Inc. (Boulder, CO, USA) initiated a phase I clinical trial (NCT02580552 [102]) to determine the efficacy and safety of anticancer LNA anti-miR-155 (MRG-106 or Cobomarsen) in patients diagnosed with mycosis fungoides, a subtype of cutaneous T-cell lymphoma (CTCL). Since cobomarsen showed an efficient reduction of the tumor burden while maintaining an acceptable safety profile during this trial, phase II clinical trials with CTCL patients (NCT03837457 [103]/NCT03713320 [104]) were initiated to assess the efficacy of cobomarsen compared to vorinostat, an FDA-approved treatment for CTCL [26].

Currently, the number of clinical trials involving lncRNA-targeting strategies is increasing; however, they are mainly focused on solid tumors. For example, the FDA approved a phase I clinical trial of Andes-1537, an ASO that targets mitochondrial lncRNAs for advanced metastatic cancer (NCT02508441 [105]/ NCT03985072 [106]).

## 6. Conclusions

Despite the best efforts to uncover the regulatory role of miRNAs and lncRNAs in lymphoma, additional research is needed to better understand the complex functional network behind miRNAs/lncRNAs regulatory interactions during lymphomagenesis in order to translate this knowledge into clinical practice. Especially regarding lncRNA biology and their function, this is still an area of investigation in its infancy.

Theoretically, given the pleiotropic function of miRNAs and lncRNAs, the target of these molecules could result in an efficient reversion of malignant phenotypes in multifactorial pathologies like lymphoma or any other cancer. In fact, the importance of miRNA/lncRNA-based therapies is highlighted by the increasing number of preclinical studies and clinical trials, especially after the groundbreaking achievement of mRNA-based anti-COVID-19 vaccines.

The field of nanomedicine is now focusing their efforts on improving the pharmacokinetics and pharmacodynamics of miRNA/lncRNA-based therapeutics. The biggest challenges in developing miRNA/lncRNA-based therapeutics are not only identifying the best ncRNA candidate but, also, to optimize the delivery vehicles. The success of this therapeutic approach depends on the development of highly stable delivery systems with low cytotoxicity and tissue-specific targeting.

## Figures and Tables

**Table 2:** miRNA and lncRNA-based therapies in clinical trials.

Drug/Therapy Agent	ClinicalTrials.Gov Identifier	Phase/Trial Status	Disease
MRX34 (miR-34 mimic)	NCT01829971 [97]	Phase I (terminated)	Primary liver cancer, NSCLC, Lymphoma, Melanoma, MM, Renal cell carcinoma
MesomiR-1 (miR-16 mimic)	NCT02369198 [99]	Phase I (completed)	Malignant pleural mesothelioma, NSCLC
MRG-106 or Cobomarsen (anti-miR-155)	NCT02580552 [102]	Phase I (completed)	CTCL (Mycosis fungoides), CLL, ABC-DLBCL, ATLL
	NCT03837457 [103]	Phase II (terminated)	CTCL (Mycosis fungoides)
	NCT03713320 [104]	Phase II (terminated)	
Andes-1537	NCT02508441 [105]	Phase I (terminated)	Advanced unresectable solid tumors
	NCT03985072 [106]	Phase I (recruiting)	Gallbladder and biliary tract carcinoma; Cervical carcinoma; Gastric carcinoma; Pancreatic carcinoma, Colorectal carcinoma.

Abbreviations: NSCLC: non-small cell lung carcinoma, MM: multiple myeloma, CTCL: cutaneous T-cell lymphoma, CLL: chronic lymphocytic leukemia, ABC-DLBCL: diffuse large B-cell lymphoma (ABC subtype), and ATLL: adult T-cell leukemia/lymphoma.
